# Lessons From Immune Checkpoint Inhibitor Trials in Hepatocellular Carcinoma

**DOI:** 10.3389/fimmu.2021.652172

**Published:** 2021-03-30

**Authors:** Raphael Mohr, Fabian Jost-Brinkmann, Burcin Özdirik, Joeri Lambrecht, Linda Hammerich, Sven H. Loosen, Tom Luedde, Münevver Demir, Frank Tacke, Christoph Roderburg

**Affiliations:** ^1^ Department of Hepatology and Gastroenterology, Charité University Medicine Berlin, Berlin, Germany; ^2^ Clinic for Gastroenterology, Hepatology and Infectious Diseases, University Hospital Düsseldorf, Düsseldorf, Germany

**Keywords:** hepatocellular carcinoma, immunotherapy, checkpoint inhibitor treatment, clinical trials, liver cirrhosis

## Abstract

The implementation of immune checkpoint inhibitors (ICI) into the clinical management of different malignancies has largely changed our understanding of cancer treatment. After having proven efficacy in different tumor entities such as malignant melanoma and lung cancer, ICI were intensively tested in the setting of hepatocellular carcinoma (HCC). Here they could achieve higher and more durable response rates compared to tyrosine-kinase inhibitors (TKI), that were sole standard of care for the last decade. Most recently, ICI treatment was approved in a first line setting of HCC, for cases not suitable for curative strategies. However, only a subset of patients benefits from ICI therapy, while others experience rapid tumor progression, worsening of liver function and poor prognosis. Efforts are being made to find immune characteristics that predict tumor responsiveness to ICI, but no reliable biomarker could be identified so far. Nevertheless, data convincingly demonstrate that combination therapies (such as dual inhibition of PD-L1 and VEGF) are more effective than the application of single agents. In this review, we will briefly recapitulate the current algorithms for systemic treatment, discuss available results from checkpoint inhibitor trials and give an outlook on future directions of immunotherapy in HCC.

## Introduction

Hepatocellular carcinoma (HCC) is the fifth most common malignancy in men (7.9% of all cancers) with 523,000 new cases per year worldwide and the seventh most common malignancy in women (6.5% of all cancers) with 226,000 new cases (1, 2). Although the incidence and prevalence in western world countries is lower compared to Asia, HCC represents a major medical and socioeconomic problem worldwide, being one of the leading causes of cancer-related deaths (2, 3).

The vast majority of HCC arises in the context of liver cirrhosis, that means in a setting of chronic inflammation and continuous liver injury. By constant induction of cell death and compensatory hyperproliferation, but also *via* provoking an immunogen microenvironment, chronic inflammation leads to a pro-carcinogenic milieu (4). Following the prevalence of major risk factors for liver cirrhosis, the incidence of HCC has been steadily increasing over the last decades. It was only most recently that a reversal of this trend was observed in western world countries (5). The increase of HCC cases in the USA and Europe in the last decades has been mainly attributed to the hepatitis C epidemic in the 1970s and 1980s. Moreover, the fast-growing number of obesity and metabolic syndrome, leading to nonalcoholic fatty liver disease (NAFLD) and steatohepatitis (NASH), is likely to condition a future increase of liver cirrhosis and also HCC - despite the foreseeable decline of hepatitis C-related HCC. In a few countries (e.g., Thailand, Japan, Singapore) the HCC incidence could be stabilized or reduced by hepatitis B vaccination programs (6).

Despite significant advances in diagnosis and tumor therapy, the prognosis of HCC remains poor, especially in advanced stages. This is particularly due to the fact that HCC often occurs in functionally compromised livers or is only diagnosed when curative therapies such as resection, transplantation, or local ablative techniques are no longer possible. These patients are left to palliative treatment options only, including systemic tumor therapy. With the introduction of tyrosine-kinase inhibitors (TKI) and recently immune checkpoint inhibitors (ICI), pharmacological treatment options for patients with advanced HCC have greatly improved. Nevertheless, their efficacy is still not satisfying. Thus, there is an unmet need for novel treatment options to further improve patients’ prognosis.

In this review, we will briefly recapitulate the current algorithms for systemic treatment, discuss available results from checkpoint inhibitor trials and give an outlook on future directions of immunotherapy in HCC.

## Current and Emerging Therapeutic Options for HCC

Continuous viral (e.g., chronic hepatitis B, C, delta co-infection), toxic or metabolic liver injury leads to chronic liver inflammation and conditions the transformation toward fibrosis and cirrhosis. The proinflammatory environment of liver cirrhosis provides an ideal breeding ground for the development of hepatocellular carcinomas.

In this context, close surveillance for all patients with cirrhosis has been recommended in international guidelines. Nevertheless, numerous primary liver tumors are still diagnosed at tumor stages that are no longer curative (intermediate or advanced stages of HCC according to the Barcelona Clinic of Liver Cancer (BCLC) staging system) (7). According to current guidelines these patients should be treated with systemic therapy. However, pharmacological treatment of HCC is challenging as HCCs show important tumor heterogeneity and arise from a distinct microenvironment, with regard to different etiologies of liver injury and different degrees of liver dysfunction. Considering the individual tumor microenvironment could be particularly relevant for immune-stimulating ICI strategies, as this might aggravate inflammatory and fibrogenic processes, e.g. in NASH (8).

HCC has long been considered to be refractory to systemic therapy. Trials with classical chemotherapy such as platinum derivatives or gemcitabine did not lead to a significant improvement in survival but proved to be very toxic against a background of impaired liver function. In 2008, the SHARP trial established sorafenib, which simultaneously inhibits tumor growth by targeting the Raf-MEK-ERK cascade as well as angiogenesis by targeting vascular endothelial growth factor (VEGFR) 2, platelet-derived growth factor receptors (PDGFR) and KIT as a novel standard treatment in patients with advanced HCC (9). Although sorafenib showed greater efficacy in certain subgroups, such as patients with hepatitis C virus infection or elevated neutrophil-lymphocyte ratio (NLR), its overall moderate efficacy and poor toxicity profile limited its use in clinical practice (10, 11). In 2018, lenvatinib, another TKI targeting VEGFR 1-3, fibroblast growth factor receptor (FGFR) 1-4, PDGFR, RET and KIT (12), was tested as non-inferior to sorafenib in the REFLECT trial and represented an alternative to the latter in the first line treatment of patients with advanced HCC or intermediate HCC refractory to loco-ablative treatments. Just recently, donafenib, another TKI was suggested as a third TKI suitable for first line therapy of HCC. In a phase II/III trial donafenib was associated with a longer median overall survival (OS) when compared with sorafenib (12.1 vs. 10.3 months, p = 0.0363), no significant differences were observed in the median progression free survival (3.7 vs. 3.6 months, p = 0.2824), objective response rate (4.6% vs. 2.7%, p = 0.2448), and disease control rate (30.8% vs. 28.7%, p = 0.5532) (13).

OS with TKI treatment was approximately one year in both the SHARP and REFLECT trials, with a progression free survival (PFS) of approximately 4 months. After disease progression under a TKI, re-administration of a TKI was tested in the RESORCE study and the CELESTIAL study, with regorafenib and cabozantinib respectively. Both substances target the VEGFR 1-3, as well as the MET and AXL pathway (14), and have been approved for use in patients refractory to sorafenib (15, 16). In addition to these classical TKI, Ramucirumab, a novel antibody directed against VEGFR 2 has demonstrated efficacy when used in patients with elevated serum alpha-fetoprotein (AFP) levels (17). In summary, TKI built the standard-of-care treatment for patients with advanced HCC or intermediate stage HCC, refractory to, or unsuitable for loco-ablative treatments. However, moderate efficacy and unfavorable toxicities conditioned the need for better treatment options.

**Figure 1:**
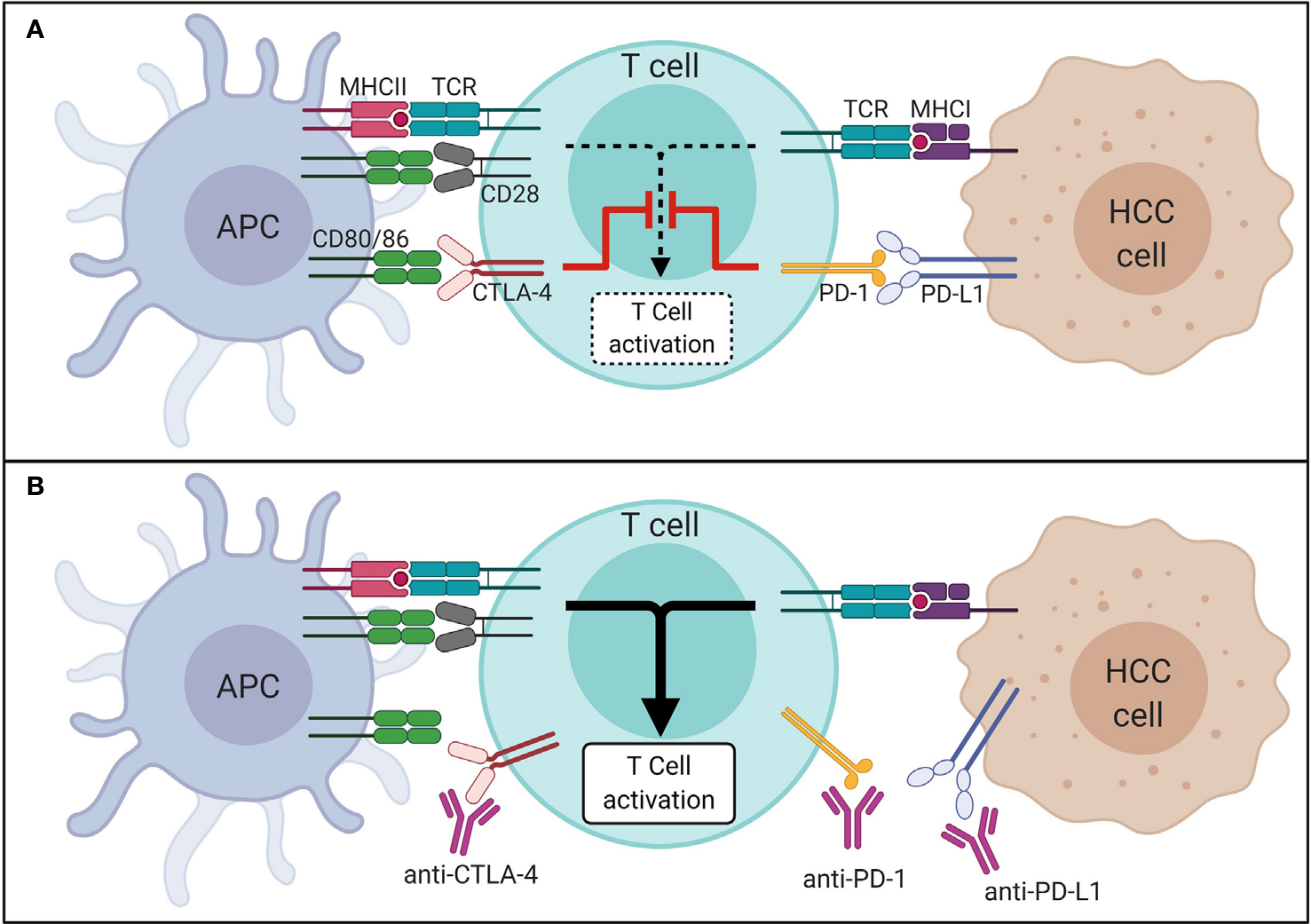
Mechanism of immune checkpoints in HCC in absence **(A)** and presence **(B)** of ICI. APC, antigen presenting cell; CTLA-4, cytotoxic T-lymphocyte-associated protein 4; HCC, hepatocellular carcinoma; ICI, immune checkpoint inhibitor; MHC, major histocompatibility complex; PD-1, programmed cell death 1 protein; PD-L1, programmed death-ligand 1; TCR, T cell receptor.

The introduction of ICI into the clinical management of different malignancies has changed our view on how to treat cancer. Immune checkpoints are “control points” of the immune system. They are based on surface receptors that, together with their ligands, prevent the immune system from attacking the body’s own cells. In many malignant tumors, proteins that target immune checkpoints are upregulated. This allows the tumor cells to escape from attacks of the immune system (immune evasion). As shown in **Figure 1**, ICI block inhibitory immune checkpoints and thus trigger a defense response of the immune system toward tumor tissue. Immunotherapies seemed promising in patients with primary liver cancer, since cirrhotic livers feature an immunosuppressive environment that protect cancer cell from being recognized by the immune system, which, in turn, may be overcome by ICI (18). ICI were and are being tested in many different studies in the context of HCC.

## Single-Agent Immunotherapy

Immunotherapy has become a new and promising pillar in the treatment of HCC. So far mainly monoclonal antibodies inhibiting programmed cell death 1 protein (PD-1), programmed death-ligand 1 (PD-L1), or cytotoxic T-lymphocyte-associated protein 4 (CTLA-4) were used in clinical trials of ICI.

Nivolumab blocks PD-1 and was tested in the setting of HCC in the non-comparative CheckMate 040 study (19). Patients with Child-Pugh A, pretreated with sorafenib (n = 182) or treatment-naïve (n = 80), received nivolumab in this phase I/II study in a dose-escalation (0.1–10 mg/kg every 2 weeks (Q2W)) and in a dose-expansion phase (3 mg/kg Q2W). Primary endpoints were safety and tolerability for the escalation phase and objective response rate (ORR) for the expansion phase. ORR and disease control rate (DCR) were 20% and 64%, respectively. 91% of responders had responses lasting 6 months or longer, and 55% had responses lasting 12 months or longer. Median OS duration was 28.6 months in sorafenib naïve patients and 15 months in patients pretreated with sorafenib. Additionally, a cohort of 49 patients with Child-Pugh B received a 240 mg flat dose of nivolumab Q2W. Interestingly, the safety profile of nivolumab in these patients was comparable to that observed in patients with Child-Pugh A. In a Child-Pugh B setting, nivolumab monotherapy also demonstrated durable responses with ORR of 10% and DCR of 55% (20).

Based on that data, phase III CheckMate 459 study compared nivolumab 240 mg Q2W (n = 371) to sorafenib (n = 372) in a first line setting. The differences in OS failed to meet statistical significance. The 33-months OS was 29% for nivolumab vs. 21% for sorafenib (21). Nevertheless, overall improvements in median OS (16.4 vs. 14.7 months), ORR (15% vs. 7%, respectively), and CR rate (4% vs. 1%, respectively) were considered clinically meaningful (22). The excellent survival in both arms is probably attributable to the subsequent therapy that patients received (49% for nivolumab and 53% for sorafenib, with 20% of patients treated with sorafenib receiving subsequent immunotherapy), which probably contributed to the study’s negative results (23). Moreover, a slower deterioration of liver function as evidenced by albumin or bilirubin levels and Child–Pugh scores was observed under nivolumab therapy.

To establish potential associations between HCC immunobiology and clinical outcomes, inflammatory gene expression signatures were assessed retrospectively from the CheckMate 040 population (24). Tumor responses were observed regardless of tumor cell PD-L1 status. Median OS was 28.1 vs. 16.6 months for patients with tumor PD-L1 ≥1% vs. <1% (p = 0.03). Tumor inflammation measured by CD3 or CD8 showed a non-significant trend toward improved OS (p = 0.08), whereas macrophage markers were not associated with OS. Tumor PD-1 and PD-L1 expression were associated with improved OS (p = 0.05 and p = 0.03, respectively). These analyses suggest that anti-tumor immune response may play a role in the treatment benefit of nivolumab in HCC.

In the keynote-224 (phase II, n = 104) and keynote-240 study (phase III, n = 413) the PD-1 inhibitor pembrolizumab was applied after sorafenib failure or intolerance. Patients received a fixed dose of 200 mg every 3 weeks (Q3W). In the phase II trial ORR (primary end point) was 18%, DCR was 61%, and OS was 12.9 months (25). The phase III trial compared pembrolizumab vs. placebo and failed to reach prespecified level of statistical significance for OS (13.9 vs. 10.6 months, respectively) and PFS (3.0 vs. 2.8 months, respectively) (26). Nevertheless, ORR was significantly higher with pembrolizumab (18% vs. 4%, p = 0.00007), and median duration of response (DOR) was 13.8 months with pembrolizumab. Survival in the sorafenib control arm was again very long, attributable to the exclusion of macrovascular invasion, better management of patients, and the availability of subsequent therapies, including immunotherapies, that were not available at trial initiation (23). While failing statistical significance, a clinical benefit of durable responses for patients who achieved a response to treatment could be demonstrated in both studies.

Along with nivolumab and pembrolizumab, camrelizumab, another PD-1 antibody, was evaluated in a phase II trial with 3 mg/kg every 2 or 3 weeks (n = 109 vs. 108, respectively). ORR was 15%, OS probability at 6 months was 74%, median OS was 13.8 months (27). Treatment-related serious adverse events occurred slightly higher in the every 2 weeks group (15% vs. 7%). Immune-related adverse events of any cause occurred in 80% in the every 2 weeks group and 87% in the every 3 weeks group. Overall, camrelizumab had a safety profile similar to other PD-1 ICIs, except for higher occurrence of reactive cutaneous capillary endothelial proliferation.

Similar results were obtained when applying durvalumab 10 mg/kg Q2W to pretreated HCC patients in a phase I/II trial (n = 40). ORR was 10%, median OS was 13.2 months (28).

In a phase Ia/Ib study tislelizumab’s dose was evaluated with 200 mg Q3W. ORR in pretreated HCC patients was 12% (29). A phase III trial is comparing tislelizumab with sorafenib in treatment naïve patients, primary endpoint is OS (NCT03412773).

## Combination Strategies for Immunotherapy

Dual blockade of PD-(L)1 and VEGF has the potential to increase antitumoral activity through joint mechanisms (30). This was the rationale for the phase 1b study assessing efficacy and safety of atezolizumab 1200 mg Q3W alone (n = 59) and combined with bevacizumab 15 mg/kg Q3W (n = 60) in a first line setting. Longer median PFS was associated with combination therapy compared to sole application of the ICI (5.6 vs. 3.4 months, p=0.011) (31). In the phase III IMbrave 150 trial a fixed dose of atezolizumab 1200 mg and bevacizumab 15 mg/kg Q3W (n* =* 336) was compared with sorafenib (n* =* 165) in a 2:1 ratio in therapy naïve patients with unresectable HCC and Child–Pugh score ≤ 6. Coprimary endpoints were OS and PFS. Underlying etiology for liver cirrhosis was predominantly viral hepatitis B and C. Macrovascular invasion was frequent and most patients were staged as BCLC C. Median PFS was 6.8 months in the combination group and 4.3 months in the sorafenib group (HR: 0.59, p < 0.0001). OS at 12 months was 67% vs. 55%, respectively. Median OS was not reached in the combination arm. Grade 3 or 4 adverse events occurred in 57% and 55%, respectively. Except for hypertension, other high-grade toxic effects were infrequent (32). Besides symptoms of impaired liver function, patients with HCC frequently suffer from diverse conditions that limit their daily lives and make systemic therapy a challenge. Against this background, the effect on patients’ quality of life is an increasingly important endpoint in the contemplation and evaluation of new therapies. The IMbrave 150 trial included the prespecified endpoints of time to deterioration (TTD) of quality of life, physical functioning, and role functioning, assessed by the European Organization for the Research and Treatment of Cancer Quality-of-Life Questionnaire (EORTC QLQ-C30). EORTC QLQ-C30 addresses these issues on a 100-point scale, with a drop of at least 10 points considered to be clinically meaningful (33). In both arms > 90% of patients completed the questionnaire, highlighting the quality of the analysis. Compared with sorafenib, the combination of atezolizumab/bevacizumab delayed TTD of patient-reported QOL (median TTD 11.2 vs. 3.6 months; physical functioning (median TTD 13.1 vs. 4.9 months), role functioning (median TTD 9.1 vs. 3.6 months)). Moreover, immunotherapy delayed TTD in patient-reported appetite loss, fatigue, pain, and diarrhea when comparing to sorafenib. A lower proportion of patients receiving the combination therapy experienced clinically meaningful deterioration in each of these symptoms when compared to TKI. In line with these results, a recent analysis demonstrated that the combination therapy showed similar efficacy regardless of age (34). In older patients, aged ≥ 65 years, the median OS was not reached in the atezolizumab/bevacizumab arm vs. 14.9 months in the sorafenib arm. In older patients PFS (7.7 vs. 4.8 months, respectively) and ORR (26% vs. 13%, respectively) also favor the application of combination therapy. Frequency and severity of adverse events were similar between the 2 age groups and consistent with the known safety profiles of atezolizumab/bevacizumab. Notably, no additional risks or toxicities were reported in older patients. Considering safety and efficacy data, these findings support an overall clinical benefit in patients with unresectable HCC. The combination of atezolizumab/bevacizumab was recently approved by European authorities and is being incorporated in guidelines as first-line therapy in advanced HCC.

Another strategy to induce a stronger immune response and enhance the clinical efficacy of ICI monotherapy, was to simultaneously block two different immune checkpoints. In the setting of non-small-cell lung carcinoma and melanoma high doses of anti CTLA-4 in combination with a PD-(L)1 inhibitor resulted in an initial proliferation and increase of peripheral T cells (35, 36). In the phase I/II CheckMate 040 trial nivolumab (anti PD-1) and ipilimumab (anti CTLA-4) were administrated in different doses and regimens to patients previously treated with sorafenib (n = 148). The primary endpoint ORR was 31% with a median DOR of 17 months. Thus, this combination led to an ORR twice that of nivolumab monotherapy. DCR was 49%, OS at 24 months was 40% (37). These results led to the currently recruiting phase III CheckMate 9DW trial, comparing nivolumab 1 mg/kg Q3W plus ipilimumab 3 mg/kg for 4 doses to sorafenib or lenvatinib in therapy naïve patients with a Child-Pugh sore ≤ 6 (NCT04039607, planned n = 1084). Primary endpoint is OS, secondary endpoints are ORR, DOR, and time to symptom deterioration (TTSD). In the phase I/II Study 22 trial patients with sorafenib failure or intolerance received durvalumab (anti PD-L1) and/or tremelimumab (anti CTLA-4) either as monotherapy or as combination therapy with different dosages (n = 40 + 332). Best median OS with 18.7 months could be achieved with the combination of a single priming dose of tremelimumab 300 mg combined with durvalumab 1500 mg being continued in a Q4W regimen, the ORR was 24% (38). Pharmacodynamic biomarker analyses showed that CD8+ lymphocyte expansion was associated with treatment response. The durvalumab/tremelimumab combination is currently being compared to sorafenib in the phase III Himalaya trial in a first line setting (NCT03298451, planned n = 1200). Primary endpoint is OS, secondary endpoints are TTP (time to progression), PFS, ORR, DCR, DOR, and safety.

A synergistic effect is expected, when combining immunotherapy and directly targeting TKIs. The phase Ibkeynote-524 trial tested lenvatinib (12 mg if ≥ 60 kg, 8 mg if < 60 kg) plus pembrolizumab 200 mg Q3W (n = 104). ORR was 46% with a median DOR of 12.6 months. Median PFS was 8.6 months (39). Based on these findings, a double-blind randomized phase III trial is comparing the combination of lenvatinib/pembrolizumab vs. lenvatinib alone in therapy naïve patients with Child-Pugh score A (NCT03713593, planned n = 750). Primary endpoints are OS and PFS, secondary endpoints are ORR, DOR, DCR, TTP, and safety.

Other combinations are being tested in phase III trials in the setting of patients with advanced HCC who did not previously receive systemic therapy, e.g., atezolizumab/cabozantinib vs. sorafenib (NCT03755791, planned n = 740), or camrelizumab/apatinib vs. sorafenib (NCT03764293, planned n = 510). In this context, another CheckMate 040 cohort compared nivolumab/cabozantinib vs. the triple combination of nivolumab/ipilimumab/cabozantinib applied in different regimens. Investigator-assessed ORR was 17% in the nivolumab/cabozantinib arm and 26% in the nivolumab/ipilimumab/cabozantinib arm. DCR was 81% vs. 83%, and median PFS was 5.5 vs. 6.8 months, respectively. Median OS was not reached in either arm. No new safety signals were observed in either arm, demonstrating than even very intensive combinations are feasible in patients with HCC (40).

As described above, the concept of combination therapies is to increase the efficacy of ICI by further stimulating the immune response, meaning to “make cold tumors hot”. Apart from pharmacological combinations, locoregional therapies (LRT) or transarterial chemoembolization (TACE) can be combination partners in this context. Besides local tumor control, they affect tumor immunity through complex mechanisms (41). LRT and TACE cause immunogenic cell death leading to the release of various tumor antigens. Moreover, they were demonstrated to enhance the number of dendritic cells in the HCC tumor microenvironment (42), leading to an increased antigen presentation and an enhanced response due to the activation of T-cells (43). Corroborating this concept, different trials are ongoing which are summarized in **Table 1**.

**Table 1:** Ongoing clinical trials of immune checkpoint inhibitors (ICI) in combination with locoregional therapies (LRT) or transarterial chemoembolization (TACE).

ICI	LRT/TACE	N	Primary Outcome	Secondary Outcome	Identifier (Name)	Phase
durvalumab ± bevacizumab	TACE	600	PFS	OS, QoL,	NCT03778957(EMERALD-1)	III
pembrolizumab + lenvatinib	TACE	950	PFS, OS	ORR, DCR, DOR, TTP, safety		III
PD-1 mAb, lenvatinib	TACE	56	ORR	PFS, TTP, DCR, DOR, OS	NCT04273100 (PLTHCC)	II
camrelizumab	TACE	60	PFS	TTP, OS, ORR, DCR, DOR, safety	NCT04483284	II
durvalumab + tremelimumab	cryoablation, RFA, TACE	50	PFS	safety	NCT02821754	II
durvalumab + tremelimumab	radiation	70	ORR	safety, OS, DCR, PFS, DOR, TTP	NCT03482102	II
durvalumab + tremelimumab	Y-90 SIRT, TACE	84	ORR	PFS, OS, safety, ORR, QoL	NCT04522544 (IMMUWIN)	II
nivolumab	TACE	49	ORR	PFS, TTP, OS, DOR, TTFS, QoL	NCT03572582(IMMUTACE)	II
nivolumab	Y-90 SIRT	40	ORR	TTR, DOR, TTP, PFS, OS, QoL, safety	NCT03033446	II
pembrolizumab	RFA, MWA, brachytherapy, TACE	30	ORR	TTR, RFS, OS, safety, biomarkers	NCT03753659	II
nivolumab	deb-TACE	14	safety	-	NCT03143270	I
pembrolizumab	TACE	26	safety	PFSR	NCT03397654	Ib

DCR, disease control rate; DOR, duration of response; MWA, microwave ablation; ORR, objective response rate; OS, overall survival; PD-1 mAb, programmed cell death 1 protein monoclonal antibody; PFS, progression free survival; PFSR, progression free survival rate; QoL, quality of life; RFA, radio frequency ablation; RFS, recurrence free survival; TTFS, time to failure of strategy; TTP, time to progression; TTR, time to response.

With autoimmune related adverse events (irAE), ICI therapy brought a novel spectrum of side effects, that was completely different than that known from chemotherapies. Risks of irAEs were widely reported as manageable and toxicity rates were generally lower than in TKI groups. Nevertheless, compared with cytotoxic agents, the possibility of identifying clinically relevant toxicity of ICI in early-phase clinical trials is relatively low (43% vs. 70%) (44). irAEs may develop long after the typical period of safety evaluation in oncology trials, and rather small sample sizes may not detect rare but life-threatening toxicity. In the IMbrave 150 study bleeding complications were observed in 7% of the atezolizumab/bevacizumab group vs. 4.5% in the sorafenib group. Although bleeding risk was not increased compared with that observed in previous anti-VEGF trials, a careful hepatologic management is necessary. The safety of ICI in the setting of advanced cirrhosis and their efficacy in different etiologies of liver injury remain open questions and need to be addressed in future trials.

## Immunotherapy in a (Neo) Adjuvant Setting

HCC resection is in most cases not a definitive cure of malignancy, as recurrence rate after hepatectomy is high (45). Tumor recurrence after HCC resection is approximately 70% within 5 years, whereas up to 50% show early recurrence within the first 2 years, which is associated with tumor characteristics such as a large tumor, an incomplete tumor capsule, and venous or microvascular invasion (46). Nevertheless, neoadjuvant or adjuvant therapies are not recommended as they have not been proven to improve the outcome. The phase III Storm trial evaluated sorafenib as an adjuvant treatment, but concluded that it is not an effective intervention in such a setting (47). Therefore, adjuvant strategies in patients with HCC remain an unmet medical need.

Characteristics of the immune contexture have been shown to correlate with recurrence and outcome. The density of CD3 and CD8 T cells in the tumor and its margins is a prognostic marker for recurrence (48). The presence of T cells and cytotoxic cells as well as the absence of macrophages and Th2 cells positively correlates with patient survival and does not differ between different etiologies and HCC stages (49). High expression of PD-L1 by tumor or immune cells is associated with a more aggressive tumor and is a predictor of recurrence (50). Altogether, there is a strong rationale for adjuvant immunotherapy and several clinical trials are investigating the role of ICI and antiangiogenic agents in an adjuvant setting. An overview of ongoing clinical trials is given in **Table 2**.

**Table 2:** Ongoing clinical trials of (neo)adjuvant immunotherapy for hepatocellular carcinoma (HCC).

ICI	Controls	Setting	Identifier (Name)	Phase
atezolizumab + bevacizumab	active surveillance	adjuvant after curative resection or ablation	NCT04102098 (IMbrave 050)	III
durvalumab ± bevacizumab	placebo	adjuvant after curative resection or ablation	NCT03847428 (EMERALD-2)	III
nivolumab	placebo	adjuvant after curative resection or ablation	NCT03383458 (CheckMate 9DX)	III
pembrolizumab	placebo	adjuvant after curative resection or ablation	NCT03867084 (KEYNOTE-937)	III
nivolumab ± ipilimumab	–	perioperative in (potentially) resectable HCC	NCT03222076	II
nivolumab + ipilimumab	–	neoadjuvant prior to resection	NCT03510871	II
nivolumab + ipilimumab	–	neoadjuvant prior to resection	NCT03682276 (PRIME-HCC)	I/II
nivolumab + cabozantinib	–	neoadjuvant prior to resection	NCT03299946	I

ICI have the potential to achieve significantly higher ORR than TKI. Better tumor responses lead to tumor size reduction and may make secondary resectability feasible. Thus, ICI based regimens may even open the field of neoadjuvant strategies for HCC. The combination of ipilimumab and nivolumab is currently being evaluated as a neoadjuvant therapy in patients undergoing hepatic resection, assessing tumor shrinkage and OS after resection (see **Table 2**).

## Conclusion and Future Directions

Although ICI monotherapy could achieve good responses in some patients, they could not demonstrate superiority to TKI based therapies. Most recently, the atezolizumab/bevacizumab combination was associated with an unparalleled benefit of survival. Thus, immunotherapy is likely to have a huge impact on the management of HCC due to the ability to produce durable and clinically relevant responses. With the combination of different agents, higher response rates and longer overall survival may be achieved. Nevertheless, a significant percentage of HCC do not respond to immunotherapy and an immunologic classification is urgently needed to guide treatment decisions. In both CheckMate 040 and CheckMate 459, PD-L1 expression was not correlated with tumor response and patient prognosis (24). Experimental markers such as circulating tumor cells, cell free DNA, miRNA have been studied in the context of HCC (51). However, these do not currently play a role in routine clinical practice.

ICI brought a novel spectrum of immune related adverse events. Dual blockade of CTLA-4 and PD-1 or PD-L1 results in enhanced toxicities, especially when higher doses of CTLA-4 inhibitors are used. Combinations with TKI or anti-VEGF carry the risk of higher toxicities in cirrhotic patients that demand a close surveillance and hepatologic management. The selection of patients is crucial regarding safety. Alternative strategies than immunotherapy may be preferred in the setting of liver transplantation or uncontrolled autoimmune disease. Nevertheless, ICI have the potential to stabilize quality of life for patients with HCC. A longer time to deterioration of health-related quality of life was demonstrated under ICI when compared to TKI (33). To reach optimal benefit of immunotherapy, biomarkers to predict response are urgently needed. Furthermore, the therapeutic sequence of different classes and the combination of available agents needs to be identified. Despite all remaining challenges, checkpoint inhibitors have already today revolutionized the treatment of HCC.

## Author Contributions

All authors drafted the manuscript and provided intellectual input. All authors contributed to the article and approved the submitted version.

## Conflict of Interest

The authors declare that the research was conducted in the absence of any commercial or financial relationships that could be construed as a potential conflict of interest.

The reviewer PS declared a past collaboration with one of the authors FT to the handling editor.

## Abbreviations

AFP, alpha-fetoprotein; BCLC, Barcelona Clinic of Liver Cancer; CTLA-4, cytotoxic T-lymphocyte-associated protein 4; DCR, disease control rate; DOR, duration of response; EORTC QLQ, European Organization for the Research and Treatment of Cancer Quality-of-Life Questionnaire; FGFR, fibroblast growth factor receptor; HCC, hepatocellular carcinoma; ICI, immune checkpoint inhibitors; irAE, immune related adverse events; LRT, locoregional therapies; NLR, neutrophil-lymphocyte ratio; NAFLD, nonalcoholic fatty liver disease; NASH, nonalcoholic steatohepatitis; ORR, objective response rate; OS, overall survival; PDGFR, platelet-derived growth factor receptors; PD-1, programmed cell death 1 protein; PD-L1, programmed death-ligand 1; PFS, progression free survival; TTD, time to deterioration; TTSD, time to symptom deterioration; TACE, transarterial chemoembolization; TKI, tyrosine-kinase inhibitors; VEGFR, vascular endothelial growth factor receptor.
